# The Prognostic Signature and Potential Target Genes of Six Long Non-coding RNA in Laryngeal Squamous Cell Carcinoma

**DOI:** 10.3389/fgene.2020.00413

**Published:** 2020-04-28

**Authors:** Shiqi Gong, Meng Xu, Yiyun Zhang, Yamin Shan, Hao Zhang

**Affiliations:** ^1^Department of Otolaryngology-Head and Neck Surgery, Ruijin Hospital, School of Medicine, Shanghai Jiao Tong University, Shanghai, China; ^2^Department of Radiation Oncology, The First Affiliated Hospital, Anhui Medical University, Hefei, China

**Keywords:** laryngeal squamous cell carcinoma, long non-coding RNA, prognostic signature, bioinformatic analysis, WGCNA

## Abstract

Studies have shown that long non-coding RNA (lncRNA) may act as the carcinogenic factor or tumor suppressor of laryngeal squamous cell carcinoma (LSCC). This study aims to identify the prognostic value and potential target protein-coding genes (PCGs) of lncRNAs in LSCC. The LSCC datasets were collected from The Cancer Genome Atlas (TCGA). Statistical and bioinformatic methods were used to establish and evaluate the prognostic model, identify the correlation between lncRNAs and clinical characteristics, and screen for PCGs co-expressed with lncRNAs. Weighted gene co-expression network analysis (WGCNA) identified PCG modules associated with clinical characteristics. The expression of lncRNAs and PCGs was analyzed using our LSCC patients by RT-qPCR. LINC02154, LINC00528, SPRY4-AS1, TTTY14, LNCSRLR, and KLHL7-DT were selected to establish the prognostic model. The overall survival (OS) of low-risk patients forecasted by the model was significantly better than high-risk patients. Receiver operating characteristic (ROC) curve and concordance index (C-index) validated the accuracy of the prognostic model. Chi-square test showed that six lncRNAs were associated with one of the clinical characteristics, i.e., gender, clinical stage, T and N stage, respectively. WGCNA identified PCG modules associated with gender, clinical stage, T and N stage. We took the intersection of the PCG modules of WGCNA, the differentially expressed PCGs between LSCC and normal samples, and the PCGs co-expressed with six lncRNAs. The intersection PCGs survival analysis showed that four PCGs, i.e., STC2, TSPAN9, SMS, and TCEA3 affected the OS of LSCC. More importantly, the differential expression of six lncRNAs and four PCGs between LSCC and normal samples was verified by our LSCC patients. In conclusion, we successfully established a prognostic model based on six-lncRNA RiskScore and initially screened the potential target PCGs of six lncRNAs for further basic and clinical research.

## Introduction

Laryngeal squamous cell carcinoma (LSCC) is one of the most common head and neck squamous cell carcinomas (Solomon et al., 2018), originating from the larynx epithelium, with high metastatic rate and poor prognosis (Bingol et al., 2016). Most LSCC patients are locally advanced when they are first diagnosed, with a 5-year survival rate of approximately 50% (Chan et al., 2018). Although surgery, radiotherapy, and chemotherapy have improved significantly over the past 20 years, the 5-year overall survival (OS) rate of LSCC has not improved significantly, especially for advanced patients (Gyawali et al., 2016). Therefore, there is an urgent need to establish new biomarkers or models for LSCC survival risk prediction to provide patients with more effective and personalized treatments.

Long non-coding RNA (lncRNA) has more than 200 nucleotides and no protein coding ability (Jiang et al., 2016). There is evidence that lncRNAs play a key role in a range of biological processes through transcriptional, post-transcriptional and epigenetic mechanisms (Quan et al., 2015). Studies have shown that lncRNA regulates mRNA through multiple patterns. First, lncRNA can directly bind to mRNA leading to the recruitment of the RNA-binding proteins (RBPs) that promote decay, the RBPs that suppress translation, or factors that initiate translation. LncRNA may also prevent miRNA from binding to target mRNA through the lncRNA-mRNA complex. Second, lncRNA can be used as miRNA sponge. By sequestering miRNAs, they reduce the availability of AGO2/RISC and relieve numerous instances of miRNA-mediated translational repression. Third, lncRNA can also serve as ‘decoys’ for RBPs, dissociating RBPs from target mRNAs, and thereby influencing the abundance and translation of such mRNAs (Noh et al., 2018). Abnormally expressed lncRNAs have been observed in various cancers including lung cancer (Seiler et al., 2017), gastric cancer (Zhuo et al., 2019), liver cancer (Noh and Gorospe, 2018), breast cancer (Wang et al., 2017), and LSCC (Wang et al., 2016; Xie et al., 2018) and so on. It has been reported that abnormally expressed lncRNAs are involved in the pathogenesis of cancer and act as a carcinogenic factor or tumor suppressor regulator in the occurrence and progression of cancer (Zhang et al., 2013; Lin and Yang, 2018). Studies have shown that lncRNAs are associated with OS in patients with LSCC (Shen et al., 2014), but the prognostic value of a single candidate lncRNA biomarker is limited. In view of this, combining a series of lncRNAs is more significant in predicting the prognosis of LSCC.

Weighted gene co-expression network analysis (WGCNA) is widely used to analyze large-scale data sets and to find modules for highly related genes (Tang et al., 2018), and it is successfully used to explore the association between gene expression information and clinical characteristics, and to identify candidate biomarkers (Langfelder and Horvath, 2008).

In this study, we identified for the first time the six-lncRNA signature as predictors of LSCC patient survival risk, using a cohort of LSCC cases from The Cancer Genome Atlas (TCGA) database. Meantime, we screened the potential target protein-coding genes (PCGs) of six lncRNAs. More importantly, we verified that six lncRNAs and four PCGs were differentially expressed in tumor tissues and adjacent normal tissues using our own 25 LSCC patients and The Human Protein Atlas database. We successfully established a prognostic model based on six-lncRNA RiskScore and initially screened the potential target PCGs of six lncRNAs for further basic and clinical research.

## Materials and Methods

### The Laryngeal Squamous Cell Carcinoma Datasets

The LSCC datasets were obtained from TCGA^1^. The database contained a total of 123 laryngeal samples, including 12 normal samples and 111 LSCC samples with clinical and gene expression data, from which the lncRNAs and PCGs were isolated. The clinical characteristics of 111 LSCC samples were shown in Supplementary Table 1.

We also collected 25 cases of LSCC patients’ tumor tissues and adjacent normal tissues from the Ruijin Hospital, School of Medicine, Shanghai Jiao Tong University. Our LSCC patients (or their parents or guardians) have signed the written informed consent form. The use of human tissue samples has been approved by the Ruijin Hospital Ethics Committee.

### Identification of Differentially Expressed lncRNAs and PCGs in LSCC

All analyses were performed using R software^2^ (version 3.5.3). The edgeR package was used to identify differentially expressed lncRNAs and PCGs between LSCC and normal samples. | log _2_ fold change (FC) | > 1 and false discovery rate (FDR) < 0.05 were set as a threshold.

### Cox Regression Analysis

RNA-seq expression values were converted by log 2 to normalize the data. The association between lncRNA expression and patient’s OS was determined by univariate Cox analysis using the Survival R package. We selected lncRNAs with *P* < 0.005 in univariate Cox analysis for multivariate Cox analysis to establish a model for predicting LSCC patient’s OS. Multivariate Cox analysis was also used to test whether RiskScore was independent of clinical parameters such as age, gender, pathological grade, clinical stage, and history of exposure to tobacco and alcohol.

### Risk Survival Curve and Model Evaluation

The RiskScore of each LSCC patient was calculated and the patient was divided into low-risk and high-risk groups using the median of RiskScore as a threshold. Kaplan–Meier survival curve was drawn for the low-risk and high-risk LSCC, and a log-rank test was used to determine the difference in OS between the two groups. The sensitivity and specificity of the six-lncRNA prognostic model were assessed by calculating the area under curve (AUC) of receiver operating characteristic (ROC) curve using the survivalROC R package, and the concordance index (C-index) using the survcomp R package.

### Establishment and Evaluation of the Nomogram

The composite nomogram for predicting OS of LSCC was established using the rms R package based on the independent risk factors from multivariate Cox analysis. The C-index was calculated using the survcomp R package to evaluate the discriminative ability of the nomogram. A calibration curve was drawn using the rms R package to compare the predicted and actual OS.

### Weighted Gene Co-expression Network Analysis (WGCNA)

The gene expression data was obtained from TCGA. A total of 16899 PCGs were identified for each sample. The variance analysis was performed, and it was ranked from large to small. The top 25% of PCGs (4225 PCGs) with larger variance were selected for WGCNA analysis.

The expression profile of 4225 PCGs was used to construct a gene co-expression network using the WGCNA package in R software (Langfelder and Horvath, 2008). An adjacency matrix was constructed using the WGCNA function adjacency by calculating the Pearson correlation between all pairs of genes in all selected samples. In this study, the power of β = 5 (scale-free *R*^2^ = 0.98) was used as a soft threshold parameter to ensure a scale-free network. To further identify functional modules in the co-expression network with 4225 PCGs, the adjacency matrix was used to calculate the topological overlap measurement (TOM) representing the overlap in the shared neighbors.

### Identification of Clinically Significant Modules

The module eigengenes (MEs) were considered to be a representation of the gene expression profile in the module. Correlation and *P*-values between the module and clinical characteristics were evaluated by calculating the MEs. In the correlation between the module and clinical characteristics, red represented positive correlation with clinical characteristics, and green represented negative correlation with clinical characteristics (Gong et al., 2019).

### Co-expression and Functional Enrichment Analysis

We tested the correlation between the expression levels of six lncRNAs and each PCGs using a two-sided Pearson correlation analysis. Identification of PCGs associated with six lncRNAs according to *P* < 0.05. Gene Ontology (GO) and Kyoto Encyclopedia of Genes and Genomes (KEGG) enrichment analysis were performed on six lncRNAs related PCGs according to *P* < 0.05 using Database for Annotation, Visualization and Integrated Discovery (DAVID, version 6.8^3^) (Huang da et al., 2009). GO analysis includes three categories: biological processes (BP), cellular components (CC), and molecular functions (MF).

### Screening the Potential Target PCGs of 6 lncRNAs

Venn diagrams were used to take the intersection of clinical significant modules of WGCNA, differentially expressed PCGs between LSCC and normal samples, and PCGs co-expressed with six lncRNAs. The intersection PCGs were divided into low expression and high expression using the median as the cut-off value. Kaplan–Meier survival curve (log-rank method) was used to evaluate the effects of PCGs on OS in LSCC patients. The Human Protein Atlas^4^ was used to validate the immunohistochemistry (IHC) of PCGs that affected OS in LSCC patients. The links to IHC images were shown in Supplementary Table 2.

### Reverse Transcription-Quantitative Polymerase Chain Reaction (RT-qPCR)

Total RNA was isolated from LSCC patients’ tumor tissues and adjacent normal tissues using TRIzol reagent (Invitrogen, Carlsbad, CA, United States). The cDNA was synthesized using HiScript III RT SuperMix for qPCR Kit (Vazyme Biotech, Nanjing, China). The cDNA was subsequently analyzed using ChamQ Universal SYBR qPCR Master Mix (Vazyme Biotech, Nanjing, China) and the ABI7500 system (Applied Biosystems, Foster City, CA, United States). The amplification program was as follows: initial denaturation step at 95°C for 30 s, followed by 40 cycles at 95°C for 5 s, and 60°C for 30 s. The expression of LINC02154, LINC00528, SPRY4-AS1, TTTY14, LNCSRLR, KLHL7-DT, STC2, TSPAN9, SMS, and TCEA3 were calculated relative to the internal reference gene, GAPDH, using the 2^–ΔΔCt^ method (Livak and Schmittgen, 2001). Primer sequences were shown in Supplementary Table 3.

### Statistical Analysis

Chi-square test was performed with SPSS (version 24.0) to identify the correlation between six lncRNAs and clinical characteristics. The results of RT-qPCR were analyzed by Graphpad Prism software (version 7.0a), and differences between groups were assessed using paired *t*-test. *P* < 0.05 was considered statistically significant.

## Results

### Differentially Expressed lncRNAs and PCGs Between LSCC and Normal Samples

According to | log _2_ FC | > 1 and FDR < 0.05, a total of 612 differentially expressed lncRNAs (Supplementary Table 4) and 4435 differentially expressed PCGs (Supplementary Table 5) were identified (LSCC compared with normal samples). Among them, 482 lncRNAs and 2516 PCGs were upregulated and 130 lncRNAs and 1919 PCGs were downregulated.

### Establishing and Evaluating a Prognostic Model Based on 6 lncRNAs

To identify lncRNA associated with prognosis, we first used a univariate Cox regression analysis to assess the association between the expression levels of each differentially expressed lncRNA and patient OS, and found that nine lncRNAs were significantly associated with OS (*P* < 0.005). Then stepwise multivariate Cox regression analysis was performed, and finally, six lncRNAs were emerged, i.e., LINC02154, LINC00528, SPRY4-AS1, TTTY14, LNCSRLR, and KLHL7-DT (Table 1). The predictive model was defined as a linear combination of expression levels of six lncRNAs whose relative coefficient weights in the multivariate Cox regression are as follows: RiskScore = (0.1779 × expression value of LINC02154) − (0.2598 × expression value of LINC00528) + (0.2075 × expression value of SPRY4-AS1) – (0.2056 × expression value of TTTY14) + (0.3098 × expression value of LNCSRLR) + (0.2924 × expression value of KLHL7-DT). Among them, LINC02154, SPRY4-AS1, LNCSRLR, and KLHL7-DT showed high-risk characteristics, and high expression means that the OS of patients was shortened. LINC00528 and TTTY14 showed low-risk characteristics, suggesting that these lncRNAs could be considered protective lncRNAs, as patients with high expression levels of these lncRNAs had longer OS than those with low expression levels.

**TABLE 1:** Multivariate Cox regression analysis to establish a prognostic model based on 6-lncRNA RiskScore.

**lncRNA**	**Multivariate Cox regression analysis**				**Differential expression of lncRNA**		
	**Coefficient**	**HR**	**SD(HR)**	***P*-value**	**logFC**	***P*-value**	**FDR**
LINC02154	0.178	1.195	0.080	0.026668	6.047	1.05E-08	1.46E-07
LINC00528	−0.260	0.771	0.117	0.026528	2.436	1.41E-05	9.14E-05
SPRY4-AS1	0.207	1.231	0.145	0.152341	2.208	7.78E-05	0.000403
TTTY14	−0.206	0.814	0.075	0.005902	−1.704	0.003552	0.010622
LNCSRLR	0.310	1.363	0.155	0.045452	1.567	0.003893	0.011445
KLHL7-DT	0.292	1.340	0.119	0.013814	1.300	0.004385	0.012654

*The differential expression of six lncRNAs (LSCC compared with normal samples). HR, hazard ratio; SD, standard deviation; FC, fold change; FDR, false discovery rate.*

**FIGURE 1:**
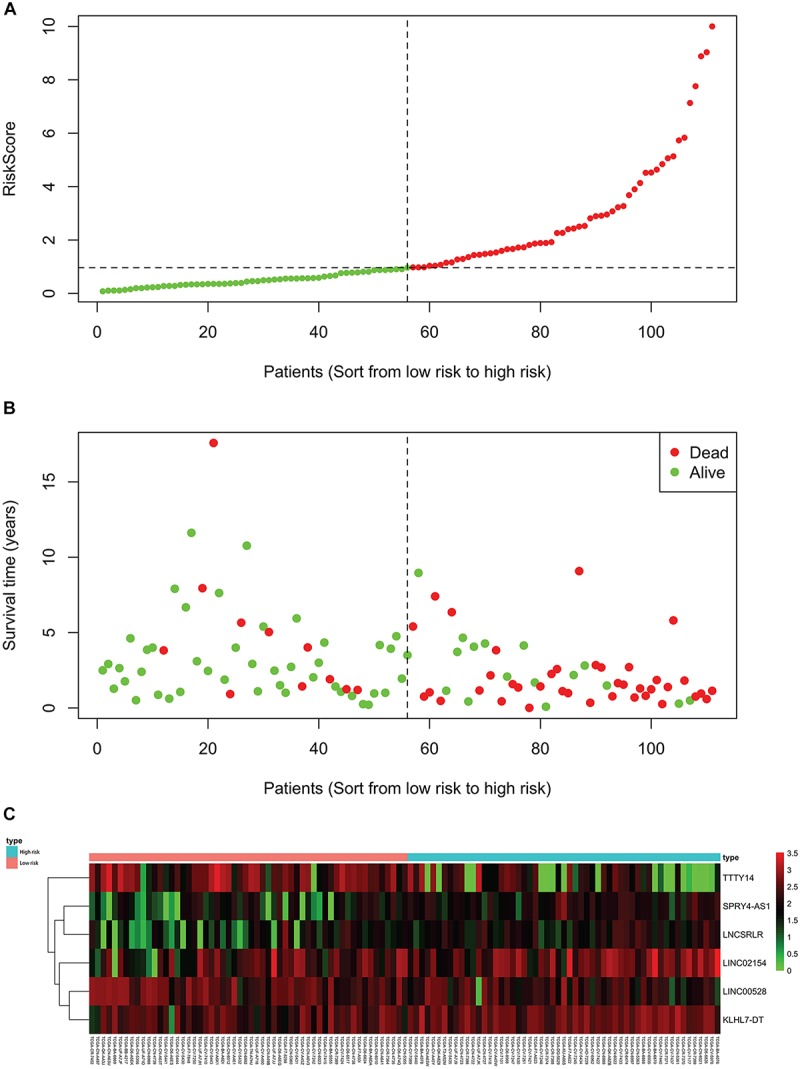
Each patient’s RiskScore, survival time and status, and the expression of six lncRNAs. The horizontal axis is based on the six-lncRNA RiskScore of patient from low to high. **(A)** RiskScore of each LSCC patient, green for low risk and red for high risk. **(B)** Survival time and status of each LSCC patient, low-risk patients to the left of the dotted line and high-risk patients to the right. **(C)** The heatmap shows the expression of six lncRNAs in each LSCC patient. The green to red indicates the expression of lncRNAs from low to high.

**FIGURE 2:**
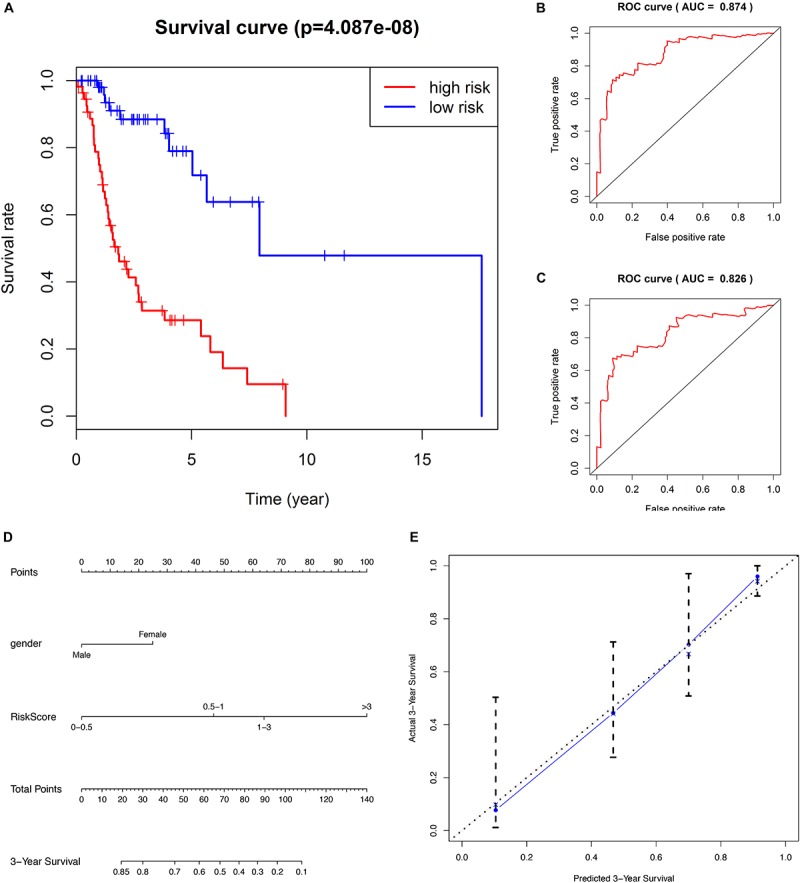
**(A)** Kaplan–Meier survival analysis indicates that the survival rate of low-risk patients was significantly higher than high-risk patients. **(B)** ROC curve of predicting 3-year survival with the AUC of 0.874. **(C)** ROC curve of predicting 5-year survival with the AUC of 0.826. **(D)** The nomogram combines patient gender with RiskScore to predict 3-year OS in patients with LSCC. **(E)** The calibration curve is used to evaluate the accuracy of the nomogram and shows good consistency between predicted survival and actual survival.

For each of the 111 LSCC patients in our study, we were able to calculate the RiskScore based on six-lncRNA expression and classify patients into low-risk (*n* = 56) and high-risk (*n* = 55) groups according to the median RiskScore of 0.964 as the cut-off value (Figure 1). Kaplan–Meier OS curve in low-risk and high-risk groups was significantly different (Figure 2A, *P* = 4.087e-08). In high-risk LSCC patients, the 3-year OS was 31.42% (95% CI: 20.38–48.40%), and the 5-year OS was 28.56% (95% CI: 17.83–45.80%). In low-risk LSCC patients, the 3-year OS was 88.40% (95% CI: 79.40–98.60%), and the 5-year OS was 79.00% (95% CI: 65.20–95.70%). The 3- and 5-year OS of low-risk LSCC were significantly higher than those of high-risk LSCC.

The predicting ability of the 6-lncRNA signature model was evaluated by calculating the AUC of the ROC curve, and the AUC of more than 0.80 was considered to be a good performance. In our study, the ROC curve of predicting 3-year survival obtained the AUC of 0.874 (Figure 2B), and the ROC curve of predicting 5-year survival obtained the AUC of 0.826 (Figure 2C), showing good sensitivity and specificity of the six-lncRNA signature model in predicting the prognosis of LSCC patients. The C-index of the model was 0.777 (95% CI: 0.71–0.835), further indicating that the model predicting the prognosis of LSCC patients has good performance.

### The Effect of 6-lncRNA RiskScore and Clinicopathological Characteristics to the Prognosis of LSCC

We evaluated the prognostic value of the clinicopathological characteristics and six-lncRNA RiskScore by univariate and multivariate Cox regression analysis. We found that female (HR, 3.428), histological grade G1+G2 (HR, 2.225) and high RiskScore (HR, 5.144) were risk factors for OS of LSCC patients. Furthermore, female (HR, 2.487) and high RiskScore (HR, 3.999) were found to be independent risk factors for OS of LSCC patients (Table 2). To facilitate the utilization of six-lncRNA RiskScore, a 3-year survival nomogram were plotted considering RiskScore and gender (Figure 2D). The C-index of the nomogram was 0.770 (95% CI: 0.704–0.836). The calibration curve for the nomogram showed good consistency between the predict and actual OS (Figure 2E).

**TABLE 2:** The prognostic value of six-lncRNA RiskScore and clinicopathologic characteristics in patients with LSCC.

**Variables**	**Univariate analysis**		**Multivariate analysis**	
	**HR (95%CI)**	***P*-value**	**HR (95%CI)**	***P-*value**
Age at initial diagnosis (>=60/<60)	0.891 (0.452,1.759)	0.740		
Gender (Female/Male)	3.428 (1.657,7.089)	0.001^∗∗^	2.487 (1.170,5.288)	0.018^∗^
Histologic grade (G1+G2/G3)	2.225 (1.009,4.908)	0.048^∗^	1.690 (0.758,3.766)	0.200
Clinical stage (IV/I+II+III)	1.006 (0.464,2.182)	0.989		
T stage (T3+T4/T1+T2)	1.438 (0.667,3.096)	0.354		
N stage (N1+N2+N3/N0)	1.897 (0.831,4.330)	0.129		
M stage (M1+Mx/M0)	1.558 (0.320,7.577)	0.583		
Alcohol history (No/Yes)	0.616 (0.333,1.139)	0.123		
Smoking history (No/Yes)	0.871 (0.457,1.659)	0.675		
6-lncRNA RiskScore (High/Low)	5.144 (2.453,10.786)	1.46E-05^∗∗∗^	3.999 (1.873,8.535)	3.40E-04^∗∗∗^

*HR, hazard ratio; CI, confidence interval. ^∗^*P* < 0.05, ^∗∗^*P* < 0.01, ^∗∗∗^*P* < 0.001.*

### Identifying the Clinical Significance of 6 lncRNAs

To explore the clinical significance of the six lncRNAs, we assessed the association between the expression levels of the six lncRNAs and the clinical characteristics of LSCC patients using chi-square test (Supplementary Table 6). We found that LINC02154 was associated with patient N stage (*P* = 0.040), LINC00528 was associated with patient T stage (*P* = 0.007), SPRY4-AS1 was associated with patient clinical stage (*P* = 0.002), TTTY14 was associated with patient gender (*P* < 0.001), LNCSRLR was associated with patient clinical stage (*P* = 0.019), and KLHL7-DT was associated with patient T stage (*P* = 0.016). These results suggested that six lncRNAs might jointly regulate the clinicopathological characteristics of LSCC. We did not find six lncRNAs associated with age at initial diagnosis, histologic grade, M stage, smoking, or drinking history.

### Weighted Gene Co-expression Network Construction and Clinically Significant Modules Identification

**FIGURE 3:**
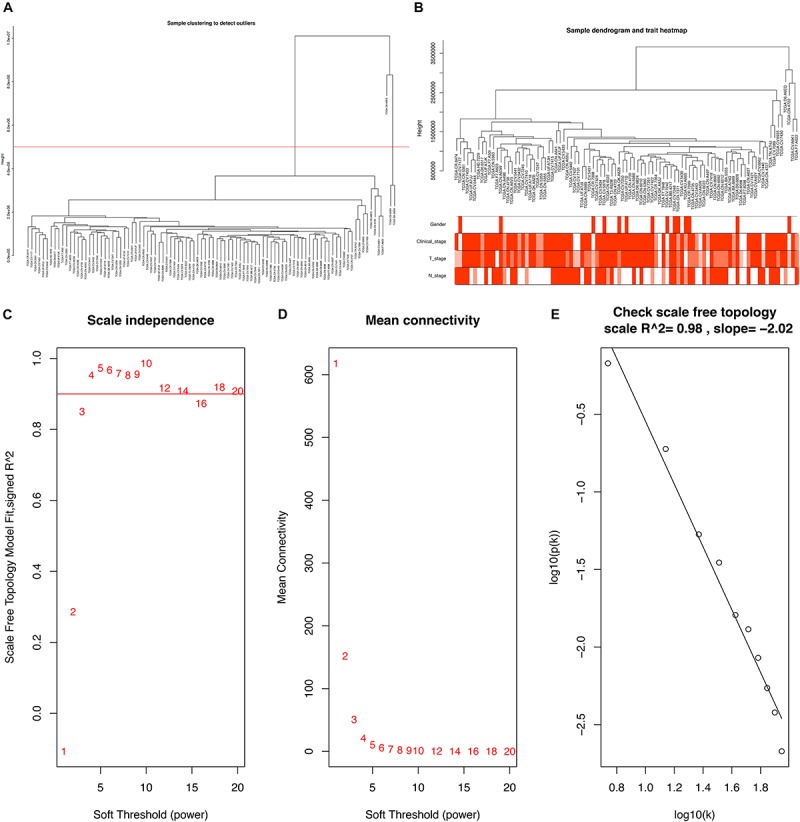
**(A)** Clustering dendrogram of 96 samples, and excluding three outlier sample. **(B)** Clustering dendrogram of 93 samples corresponding to clinical characteristics. **(C)** Analysis of the scale-free fit index for various soft-thresholding powers (β). **(D)** Analysis of the mean connectivity for various soft-thresholding powers. **(E)** Checking the scale free topology when β = 5.

To further explore the association between PCGs and clinical characteristics (gender, clinical stage, T stage, and N stage) of LSCC patient, we performed a WGCNA analysis. 15 LSCC samples were excluded due to lack of one or more of gender, clinical stage, T stage or N stage, and 96 LSCC samples were used for WGCNA analysis. The samples of LSCC (*n* = 96) were clustered using average linkage method and Pearson correlation method. We excluded three outlier sample and finally included 93 samples for subsequent analysis (Figures 3A,B).

Constructing a WGCNA needed the best soft-thresholding power to which co-expression similarity was raised to calculate adjacency. Therefore, we performed a network topology analysis of various soft-thresholding powers to have relatively balanced scale independence and average connectivity of WGCNA. In this study, the power of β = 5 (scale-free *R*^2^ = 0.98) was selected as the soft-thresholding parameter to ensure a scale-free network (Figures 3C–E).

**FIGURE 4:**
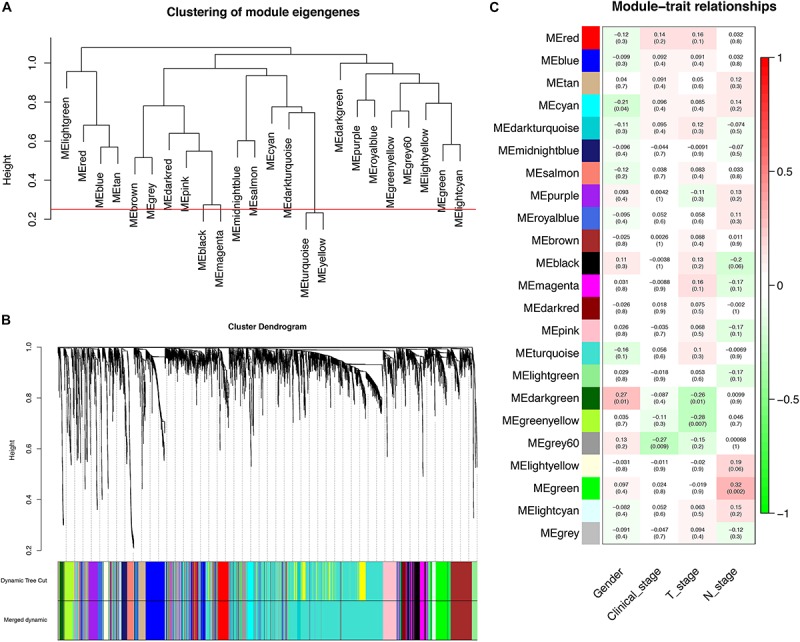
Identification of PCGs modules associated with the clinical characteristics of LSCC. **(A)** The horizontal line defined the threshold, so 23 distinct gene modules were identified. **(B)** Dendrogram of all genes were clustered based on a dissimilarity measure (1-TOM). **(C)** Heatmap of the correlation between module eigengenes (MEs) and clinical characteristics of LSCC.

Through dynamic tree cut and merged dynamics, 24 different gene modules were generated in a hierarchical clustering tree from 93 samples, and each module marked by a different color was displayed through a tree diagram, wherein each tree branch constituted a module and each leaf in the branch was one gene. As shown in Figure 4A, the horizontal line defined the threshold, by merging similar modules, 23 distinct gene modules were identified (Figure 4B). According to the standard with minimum *P*-value, we found that gender was associated with the darkgreen module (*P* = 0.010), clinical stage was associated with the grey60 module (*P* = 0.009), T stage was associated with the greenyellow module (*P* = 0.007), N stage was associated with the green module (*P* = 0.002), and those modules were selected as the clinically significant modules for further analysis (Figure 4C). The list of PCGs for clinically significant modules was shown in Supplementary Table 7.

### Co-expression Predicting PCGs Associated With 6 lncRNAs and Characterizing Their Functions

**FIGURE 5:**
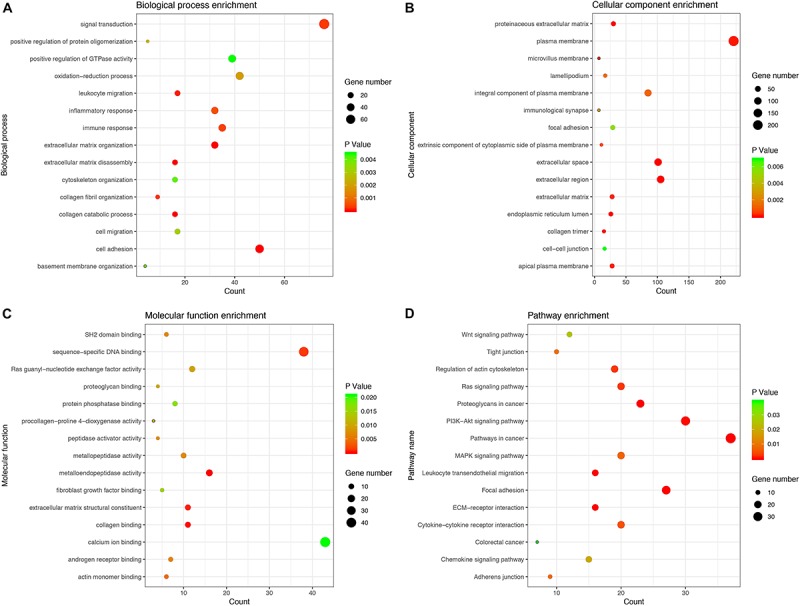
GO and KEGG enrichment analysis for PCGs co-expressed with six lncRNAs. **(A)** Biological process. **(B)** Cellular component. **(C)** Molecular function. **(D)** KEGG pathway.

We calculated the Pearson correlation coefficients between the PCGs and the six lncRNAs to determine the co-expression relationship, respectively. The PCGs with *P* < 0.05 were considered to be associated with six lncRNAs (Supplementary Table 8). To more accurately predict the potential function of the six lncRNAs, we selected 850 key PCGs with | Pearson correlation coefficient | > 0.40 and *P* < 0.001 for GO and KEGG pathway enrichment analysis. The key PCGs in the BP group were mainly enriched in extracellular matrix organization, cell adhesion, collagen catabolic process, and so on. The key PCGs in the CC group were significantly enriched in extracellular space, endoplasmic reticulum lumen, proteinaceous extracellular matrix and so on. The key PCGs in the MF group were mainly enriched in metalloendopeptidase activity, collagen binding, extracellular matrix structural constituent and so on. According to KEGG pathway analysis, the key PCGs were mainly involved in focal adhesion, pathways in cancer, proteoglycans in cancer and so on. These results indicated that the key PCGs co-expressed with six lncRNAs might be associated with the occurrence and progression of tumors (Figure 5).

### Screening the Potential Target PCGs of 6 lncRNAs

**FIGURE 6:**
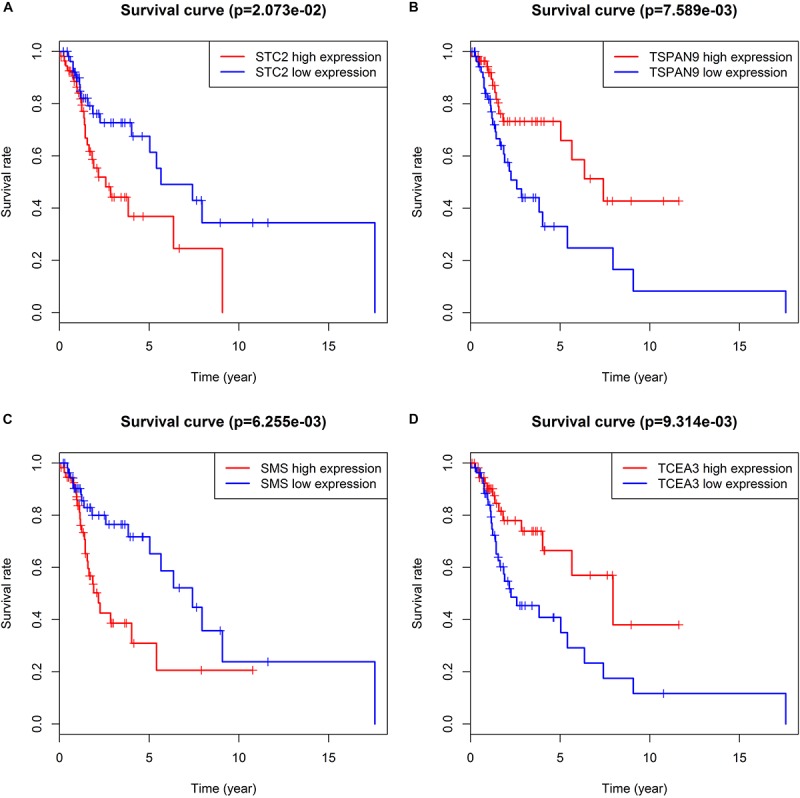
Kaplan–Meier survival analysis indicates that patients with high expression of STC2 **(A)** and SMS **(C)**, low expression of TSPAN9 **(B)** and TCEA3 **(D)** were shorter in OS compared to patients with low expression of STC2 and SMS, high expression of TSPAN9 and TCEA3.

**FIGURE 7:**
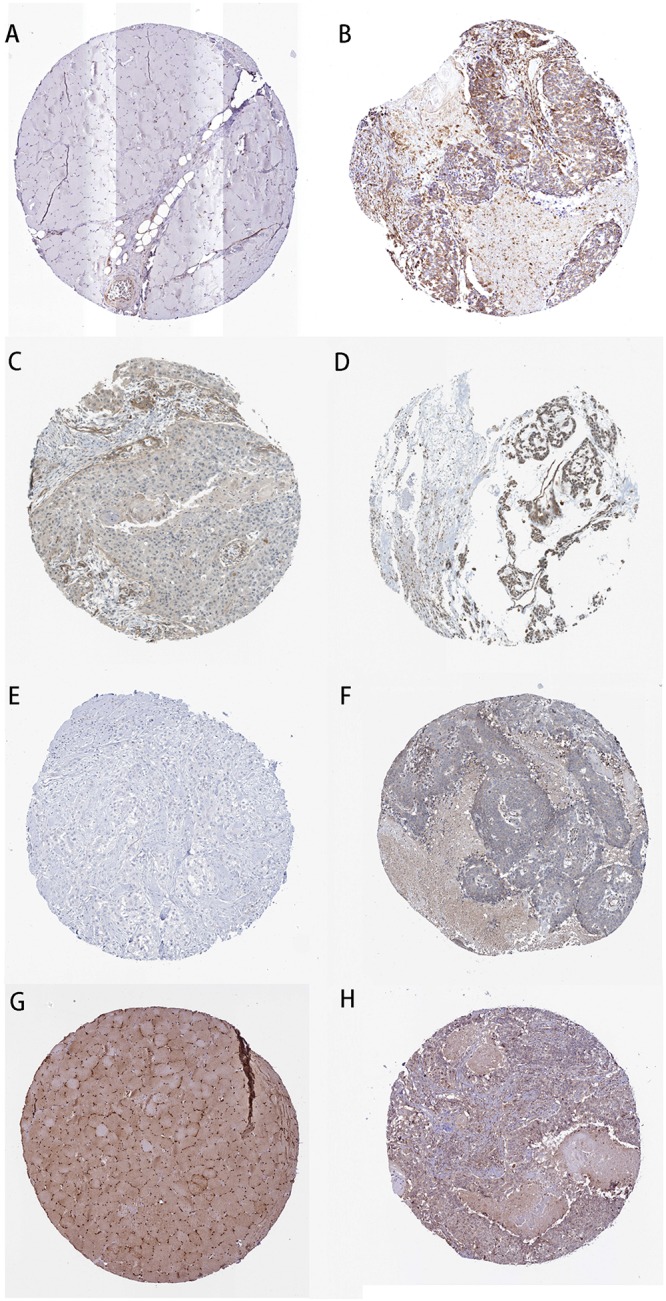
Immunohistochemistry of the four PCGs affecting OS of LSCC patients based on The Human Protein Atlas. **(A)** Protein levels of STC2 in normal tissue (staining: not detected; intensity: negative; quantity: negative). **(B)** Protein levels of STC2 in tumor tissue (staining: medium; intensity: moderate; quantity: > 75%). **(C)** Protein levels of TSPAN9 in normal tissue (staining: low; intensity: weak; quantity: 25–75%). **(D)** Protein levels of TSPAN9 in tumor tissue (staining: medium; intensity: moderate; quantity: > 75%). **(E)** Protein levels of SMS in normal tissue (staining: not detected; intensity: negative; quantity: negative). **(F)** Protein levels of SMS in tumor tissue (staining: low; intensity: weak; quantity: < 25%). **(G)** Proteins level of TCEA3 in normal tissue (staining: high; intensity: strong; quantity: > 75%). **(H)** Protein levels of TCEA3 in tumor tissue (staining: not detected; intensity: weak; quantity: < 25%).

We took the intersection of the green module of WGCNA, differentially expressed PCGs between normal samples and LSCC, and PCGs co-expressed with LINC02154. And 23 PCGs were screened as potential target PCGs for LINC02154. We took the intersection of the greenyellow module of WGCNA, differentially expressed PCGs between normal samples and LSCC, and PCGs co-expressed with LINC00528. And 15 PCGs were screened as potential target PCGs for LINC00528. We took the intersection of the grey60 module of WGCNA, differentially expressed PCGs between normal samples and LSCC, and PCGs co-expressed with SPRY4-AS1. And one PCG was screened as a potential target PCG for SPRY4-AS1. We took the intersection of the darkgreen module of WGCNA, differentially expressed PCGs between normal samples and LSCC, and PCGs co-expressed with TTTY14. And three PCGs were screened as potential target PCGs for TTTY14. We took the intersection of the grey60 module of WGCNA, differentially expressed PCGs between normal samples and LSCC, and PCGs co-expressed with LNCSRLR. And two PCGs were screened as potential target PCGs for LNCSRLR. We took the intersection of the greenyellow module of WGCNA, differentially expressed PCGs between normal samples and LSCC, and PCGs co-expressed with KLHL7-DT. And seven PCGs were screened as potential target PCGs for KLHL7-DT (Table 3). Survival analysis of potential target PCGs for six lncRNAs showed that STC2 (*P* = 0.021), TSPAN9 (*P* = 0.008), SMS (*P* = 0.006), and TCEA3 (*P* = 0.009) affected the OS of LSCC (Figure 6 and Table 3). High expression of STC2 and SMS, low expression of TSPAN9 and TCEA3 patients were shorter in OS compared to patients with low expression of STC2 and SMS, high expression of TSPAN9 and TCEA3. To further validate the differential expression of four potential target PCGs affecting OS of LSCC patients between normal samples and LSCC, we used The Human Protein Atlas database to find IHC images. We found that STC2, TSPAN9, SMS were high expression in LSCC, and TCEA3 was low expression in LSCC (Figure 7).

**TABLE 3:** The potential target PCGs of six lncRNAs, including Pearson correlation analysis between six lncRNAs and PCGs, differential expression of PCGs (LSCC compared with normal samples), and Kaplan–Meier survival analysis (log-rank method) of PCGs.

**lncRNA**	**Pearson correlation analysis**			**Differential expression of PCGs**			**Survival *P*-value**
	**PCGs**	**Cor**	***P*-value**	**logFC**	***P*-value**	**FDR**	
LINC02154	AKR1B1	0.233	0.009575	1.030	0.002855	0.008849	0.776012
LINC02154	ALDH3A1	−0.215	0.017196	−2.237	2.47E-06	1.97E-05	0.776116
LINC02154	ATP2B1	0.183	0.042640	1.160	0.000358	0.001501	0.245751
LINC02154	FADS1	0.390	8.12E-06	3.083	8.95E-14	3.08E-12	0.052014
LINC02154	FADS2	0.207	0.021816	3.094	1.21E-09	2.01E-08	0.518103
LINC02154	FBN2	0.356	5.34E-05	5.073	1.10E-08	1.51E-07	0.434999
LINC02154	FTL	0.262	0.003363	1.232	5.25E-05	0.000286	0.511502
LINC02154	GPNMB	0.302	0.000687	1.853	6.69E-06	4.73E-05	0.875062
LINC02154	GSTA1	−0.222	0.013576	−1.576	0.019205	0.043548	0.700020
LINC02154	KIAA1549L	0.237	0.008335	1.555	0.009496	0.024180	0.562459
LINC02154	MANSC1	−0.303	0.000660	−1.399	5.88E-07	5.50E-06	0.145584
LINC02154	MYH14	−0.231	0.010171	−1.259	5.20E-05	0.000284	0.598905
LINC02154	NDRG4	0.273	0.002260	1.274	0.007745	0.020398	0.887394
LINC02154	NDUFS1	−0.330	0.000190	−1.034	1.29E-12	3.65E-11	0.094758
LINC02154	PGD	−0.259	0.003761	−1.243	1.38E-05	8.96E-05	0.536211
LINC02154	RGMA	−0.232	0.009891	−1.203	0.000595	0.002330	0.781241
LINC02154	SEL1L3	0.214	0.017372	1.008	0.000550	0.002174	0.295679
LINC02154	SLC1A4	0.178	0.048385	1.326	5.97E-07	5.57E-06	0.751310
LINC02154	SLC25A36	0.179	0.047966	1.068	3.94E-07	3.86E-06	0.936942
LINC02154	SLC7A11	0.188	0.036840	1.933	0.001989	0.006479	0.561165
LINC02154	SOST	0.306	0.000572	8.075	1.55E-08	2.07E-07	0.072667
LINC02154	SPP1	0.402	4.11E-06	5.081	1.63E-08	2.17E-07	0.366869
LINC02154	STC2	0.471	3.90E-08	3.868	1.83E-12	5.04E-11	0.020726^∗^
LINC00528	ATP13A5	0.257	0.004132	2.880	0.017906	0.041045	0.557578
LINC00528	COL4A5	0.186	0.039882	2.223	1.41E-09	2.32E-08	0.221489
LINC00528	COL4A6	0.265	0.003057	2.909	3.57E-07	3.53E-06	0.705367
LINC00528	CXCL14	0.263	0.003242	2.592	0.000117	0.000574	0.100379
LINC00528	IGFBP2	0.255	0.004425	1.135	0.021610	0.047913	0.825089
LINC00528	ISYNA1	0.312	0.000437	1.544	4.06E-05	0.000228	0.990948
LINC00528	ITM2C	0.251	0.005113	1.503	6.43E-10	1.11E-08	0.779122
LINC00528	MTCL1	0.205	0.023062	1.777	1.82E-05	0.000113	0.525657
LINC00528	NDC80	0.325	0.000244	1.213	0.000196	0.000901	0.732933
LINC00528	PADI3	0.296	0.000902	2.391	0.001807	0.005963	0.870301
LINC00528	RAB3B	0.289	0.001189	5.235	6.34E-08	7.42E-07	0.575784
LINC00528	SCD5	0.356	5.29E-05	1.548	6.81E-06	4.80E-05	0.320449
LINC00528	SERPINI1	0.423	1.11E-06	2.724	0.000397	0.001647	0.524923
LINC00528	TSPAN9	0.274	0.002158	1.339	3.15E-08	3.92E-07	0.007589^∗∗^
LINC00528	TYMS	0.385	1.09E-05	1.235	0.000104	0.000519	0.884710
SPRY4-AS1	SCNN1A	−0.181	0.045419	−1.609	7.72E-07	7.00E-06	0.172846
TTTY14	NOTUM	−0.329	0.000200	5.402	1.13E-06	9.87E-06	0.319919
TTTY14	MEST	−0.307	0.000557	2.178	1.64E-08	2.18E-07	0.627868
TTTY14	SMS	−0.216	0.016482	1.100	9.49E-06	6.43E-05	0.006255^∗∗^
LNCSRLR	SCNN1A	−0.373	2.16E-05	−1.609	7.72E-07	7.00E-06	0.172846
LNCSRLR	ZBTB7C	−0.309	0.000499	−1.278	0.000406	0.001680	0.825610
KLHL7-DT	ATP13A5	−0.196	0.029812	2.880	0.017906	0.041045	0.557578
KLHL7-DT	COL4A5	0.237	0.008280	2.223	1.41E-09	2.32E-08	0.221489
KLHL7-DT	COL4A6	0.189	0.036342	2.909	3.57E-07	3.53E-06	0.705367
KLHL7-DT	CXCL14	−0.216	0.016490	2.592	0.000117	0.000574	0.100379
KLHL7-DT	GPT2	−0.254	0.004573	−1.897	6.50E-16	3.06E-14	0.663780
KLHL7-DT	LGALS3	−0.182	0.044249	−1.209	3.98E-09	6.01E-08	0.317182
KLHL7-DT	TCEA3	−0.283	0.001501	−1.441	2.21E-08	2.84E-07	0.009314^∗∗^

*PCGs, protein-coding genes; Cor, correlation coefficient; FC, fold change; FDR, false discovery rate. ^∗^*P* < 0.05, ^∗∗^*P* < 0.01.*

### Validation of the Differential Expression of the 6 lncRNAs and 4 PCGs

**FIGURE 8:**
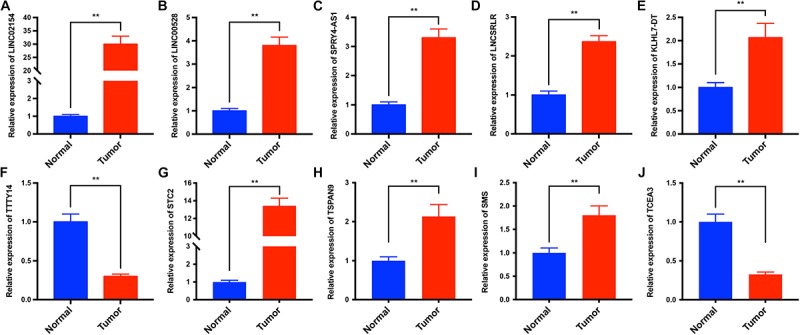
The expression levels of LINC02154, LINC00528, SPRY4-AS1, TTTY14, LNCSRLR, KLHL7-DT, STC2, TSPAN9, SMS, and TCEA3 in tumor tissues and adjacent normal tissues of 25 LSCC patients were analyzed by RT-qPCR. The results showed that the expression levels of LINC02154 **(A)**, LINC00528 **(B)**, SPRY4-AS1 **(C)**, LNCSRLR **(D)**, KLHL7-DT **(E)**, STC2 **(G)**, TSPAN9 **(H)**, and SMS **(I)** in tumor tissues were higher than those in adjacent normal tissues, and the expression levels of TTTY14 **(F)** and TCEA3 **(J)** in tumor tissues were lower than that in adjacent normal tissues (**P < 0.01).

To verify the differential expression of the six lncRNAs and four PCGs obtained from the analysis of TCGA datasets, we used RT-qPCR to analyze the expression levels of the six lncRNAs and four PCGs in tumor tissues and adjacent normal tissues of 25 LSCC patients in our hospital. The results showed that the expression levels of LINC02154, LINC00528, SPRY4-AS1, LNCSRLR, KLHL7-DT, STC2, TSPAN9, and SMS in tumor tissues were higher than those in adjacent normal tissues, and the expression level of TTTY14 and TCEA3 in tumor tissues was lower than that in adjacent normal tissues (*P* < 0.01, Figure 8). The experiment results validated the differential expression of the six lncRNAs and four PCGs we found in TCGA database.

## Discussion

Currently, the predictive indicator of prognosis in patients with LSCC still needs further exploration. Clinical TNM stage and histopathological grade are commonly used indicators to predict the prognosis of patients with LSCC (Almadori et al., 2005). Studies have shown that exposure to tobacco and alcohol and human papillomavirus infection are risk factors for prognosis in LSCC patients (Stelow et al., 2010; Nogueira et al., 2015). Abnormal expression of various PCGs and miRNAs are associated with prognosis in patients with LSCC (Pich et al., 2004; Ayaz et al., 2013; Wong et al., 2016). LncRNA is widely involved in cancer pathways (Schmitt and Chang, 2016) and is an emerging biomarker and potential therapeutic target for tumors (Chandra Gupta and Nandan Tripathi, 2017), which is closely related to the progression of various tumors (Tsai et al., 2011) and the prognosis of cancer patients (Serghiou et al., 2016). However, there is little research on the correlation between lncRNAs and prognosis in patients with LSCC.

In this study, we used multivariate Cox regression to establish a model based on six-lncRNA (LINC02154, LINC00528, SPRY4-AS1, TTTY14, LNCSRLR, and KLHL7-DT) RiskScore to predict the prognosis of patients with LSCC. The OS of low-risk patients forecasted by the model was significantly better than high-risk patients. The AUC of the ROC curve showed the good performance of the model in predicting the 3- and 5-year OS of patients with LSCC. C-index further demonstrated the accuracy of the model. Multivariate Cox analysis showed that female and high RiskScore were independent risk factors for prognosis in LSCC patients. We constructed a nomogram that combined patient gender and RiskScore. The C-index and the calibration curve confirmed the accuracy of the nomogram. This makes it easier and more intuitive to predict the 3-year OS of patients with LSCC based on patient gender and RiskScore. Chi-square test showed that six lncRNAs were associated with one of the clinical characteristics, i.e., gender, clinical stage, T stage, and N stage, respectively, indicating that six lncRNAs were involved in the regulation of the clinical characteristics of LSCC.

Studies have shown that LINC02154 is significantly upregulated in renal cell carcinoma and its high expression is one of the risk factors for poor prognosis. It is involved in the construction of a model for predicting the prognosis of patients with renal cell carcinoma (Zuo et al., 2018). TTTY14 has been known to be downregulated in oral squamous cell carcinoma. Patients with high expression of TTTY14 have a longer survival time. Multivariate Cox analysis has shown that low expression of TTTY14 is an independent risk factor for prognosis in patients with oral squamous cells (Li et al., 2017). TTTY14 is also significantly downregulated in gastric cancer, and its low expression is one of the risk factors for poor prognosis. It participates in the construction of a model for predicting the prognosis of patients with gastric cancer (Miao et al., 2017). It has been found that LNCSRLR is upregulated in renal cell carcinoma patients with intrinsic sorafenib resistance. Highly expressed LNCSRLR directly binds to NF-κB and promotes IL-6 transcription, leading to activation of STAT3 and development of sorafenib resistance (Xu et al., 2017). LNCSRLR is involved in the construction of a model for predicting the prognosis of patients with cervical squamous cell carcinoma, and its high expression is one of the risk factors for poor prognosis (Mao et al., 2019). The results of these studies are consistent with our results, LINC02154 and LNCSRLR are risk factors for prognosis in patients with LSCC, and TTTY14 is a protective factor. No studies concerning the roles of other three lncRNAs in tumors were reported.

To further explore the association between clinical characteristics and PCGs, we performed a WGCNA, which identified PCGs modules associated with patient gender (darkgreen), clinical stage (grey60), T stage (greenyellow), and N stage (green). We took the intersection of clinically significant modules of WGCNA, differentially expressed PCGs between LSCC and normal samples, and PCGs co-expressed with six lncRNAs. The intersection PCGs survival analysis showed that STC2, TSPAN9, SMS, and TCEA3 affected the OS of LSCC. GO and KEGG enrichment indicated that PCGs co-expressed with six lncRNAs might be associated with the occurrence and progression of tumors. The images of IHC from The Human Protein Atlas database indicated that STC2, TSPAN9, SMS were high expression in LSCC, and TCEA3 was low expression in LSCC. More importantly, we analyzed the expression levels of the six lncRNAs and four PCGs in our own 25 LSCC patients between tumor tissues and adjacent normal tissues by RT-qPCR. The results showed that the six lncRNAs and four PCGs were differentially expressed between tumor tissues and adjacent normal tissues, supporting the analysis results from TCGA datasets.

Studies have shown that high expression of STC2 promotes hepatocellular carcinoma proliferation (Wu et al., 2017) and induces drug resistance, resulting in poor prognosis (Cheng et al., 2018). The expression of STC2 is closely related to the prognosis of tumor patients, and its high expression leads to poor prognosis in patients with breast cancer (Esseghir et al., 2007), nasopharyngeal carcinoma (Lin et al., 2014), colorectal cancer (Ieta et al., 2009), and renal cell carcinoma (Meyer et al., 2009). STC2 protein expression in LSCC tissues is associated with invasion into the thyroid cartilage, T stage, lymphatic metastasis, clinical stage, and pathological differentiation. Circulating STC2 mRNA is potentially useful blood markers, and STC2 protein may be a prognostic marker for poor outcome following surgery in LSCC (Zhou et al., 2014). These studies indicate that the high expression of STC2 is a risk factor for the prognosis of a variety of tumor including LSCC. TSPAN9 is lowly expressed in gastric cancer. Experimental studies have shown that TSPAN9 inhibits proliferation, migration, and invasion of gastric cancer cells through the ERK1/2 pathway (Li et al., 2016). This result indicates that TSPAN9 is a tumor suppressor. Polyamine metabolism abnormalities are often present in cancer cells. Multiple abnormalities in the control of polyamine metabolism and uptake may be responsible for increased levels of polyamines in cancer cells as compared to that of normal cells, and spermine synthase (SMS) is a member of the polyamine metabolic pathway. Treatment with an SMS inhibitor can be attempted in cancer (Thomas and Thomas, 2003). The SMS inhibitor showed a strong inhibitory effect on the growth of P388 leukemia cells (He et al., 1995). SMS inhibitors can significantly inhibit tumor cell growth, so SMS may be an oncogene. TCEA3 expression is significantly downregulated in gastric cancer tissues. Poor prognoses are observed in the low TCEA3 expression group compared to the high TCEA3 expression group. Functionally, upregulation of TCEA3 inhibits gastric cancer cell proliferation and colony formation, which may attenuate cell growth through apoptosis induction (Li et al., 2015). This result indicates that TCEA3 is a tumor suppressor. The above studies are consistent with our analysis. STC2 and SMS are risk factors, and TSPAN9 and TCEA3 are protective factors.

## Conclusion

We successfully establish a prognostic model based on six-lncRNA RiskScore that effectively predicts the prognoses of patients with LSCC. This model helps risk stratification and provides more effective and personalized treatment for each patient. We initially analyzed the potential functions of six lncRNAs and screened the potential target PCGs of six lncRNAs. In the future, we will perform clinical studies to verify the predictive effects of the six-lncRNA prognostic model, and experimental studies to investigate the potential mechanisms of the six lncRNAs.

## Data Availability Statement

The datasets used for this study can be found in TCGA, https://portal.gdc.cancer.gov/.

## Ethics Statement

The use of human tissue samples has been approved by the Ruijin Hospital Ethics Committee. The patients (or their parents or guardians) have signed the written informed consent form.

## Author Contributions

SG responsible for research design, data collection, bioinformatic analysis, RT-qPCR assay, and manuscript writing. MX responsible for research design, data collection, statistical analysis, and RT-qPCR assay. YZ responsible for data organization. YS guides research ideas, design and data interpretation. HZ guides research ideas, design, research methods, and manuscript revision.

## Conflict of Interest

The authors declare that the research was conducted in the absence of any commercial or financial relationships that could be construed as a potential conflict of interest.

## Footnotes

1 https://portal.gdc.cancer.gov/

2 https://www.r-project.org/

3 https://david.ncifcrf.gov/

4 https://www.proteinatlas.org/
