# Supplementary material for: The Prognostic Signature and Potential Target Genes of Six Long Non-coding RNA in Laryngeal Squamous Cell Carcinoma
Source: Front Genet. 2020 Apr 28;11:413. doi: 10.3389/fgene.2020.00413 (PMC7198905; doi:10.3389/fgene.2020.00413)
Supplement: Supplementary file 1 [file Table_1.DOCX]

**Supplementary Table 1.** The clinicopathological characteristics of 111 LSCC patients from the TCGA database.

| Characteristics | Patients (n=111) | |
| --- | --- | --- |
|  | n | % |
| Age at initial diagnosis | |  |
| <60 | 38 | 34.23 |
| >=60 | 73 | 65.77 |
| Gender |  |  |
| Male | 91 | 81.98 |
| Female | 20 | 18.02 |
| Histologic grade |  |  |
| G1+G2 | 78 | 70.27 |
| G3 | 29 | 26.13 |
| Gx | 4 | 3.60 |
| Clinical stage |  |  |
| I+II+III | 25 | 22.52 |
| IVA+IVB+IVC | 71 | 63.97 |
| NA | 15 | 13.51 |
| T stage |  |  |
| T1+T2 | 19 | 17.12 |
| T3+T4a | 79 | 71.17 |
| Tx | 11 | 9.91 |
| NA | 2 | 1.80 |
| N stage |  |  |
| N0 | 40 | 36.04 |
| N1-3 | 52 | 46.85 |
| Nx | 17 | 15.31 |
| NA | 2 | 1.80 |
| M stage |  |  |
| M0 | 40 | 36.04 |
| M1 | 1 | 0.90 |
| Mx | 8 | 7.21 |
| NA | 62 | 55.85 |
| Alcohol history |  |  |
| Yes | 70 | 63.06 |
| No | 39 | 35.14 |
| NA | 2 | 1.80 |
| Smoking history |  |  |
| Yes | 55 | 49.55 |
| No | 53 | 47.75 |
| NA | 3 | 2.70 |

NA, Not Applicable.
