# Supplementary material for: The Prognostic Signature and Potential Target Genes of Six Long Non-coding RNA in Laryngeal Squamous Cell Carcinoma
Source: Front Genet. 2020 Apr 28;11:413. doi: 10.3389/fgene.2020.00413 (PMC7198905; doi:10.3389/fgene.2020.00413)
Supplement: Supplementary file 2 [file Table_2.DOCX]

**Supplementary Table 2.** The links to IHC images of STC2, TSPAN9, SMS, and TCEA3 in The Human Protein Atlas.

| PCGs | Tissue type | Link |
| --- | --- | --- |
| STC2 | Normal | <https://www.proteinatlas.org/ENSG00000113739-STC2/tissue/skeletal+muscle> |
|  | Tumor | <https://www.proteinatlas.org/ENSG00000113739-STC2/pathology/tissue/head+and+neck+cancer> |
| TSPAN9 | Normal | <https://www.proteinatlas.org/ENSG00000011105-TSPAN9/pathology/tissue/head+and+neck+cancer> |
|  | Tumor | <https://www.proteinatlas.org/ENSG00000011105-TSPAN9/pathology/tissue/head+and+neck+cancer> |
| SMS | Normal | <https://www.proteinatlas.org/ENSG00000102172-SMS/pathology/tissue/head+and+neck+cancer> |
|  | Tumor | <https://www.proteinatlas.org/ENSG00000102172-SMS/pathology/tissue/head+and+neck+cancer> |
| TCEA3 | Normal | <https://www.proteinatlas.org/ENSG00000204219-TCEA3/tissue/skeletal+muscle> |
|  | Tumor | <https://www.proteinatlas.org/ENSG00000204219-TCEA3/pathology/tissue/head+and+neck+cancer> |

PCGs, protein-coding genes.
