# Supplementary material for: The Prognostic Signature and Potential Target Genes of Six Long Non-coding RNA in Laryngeal Squamous Cell Carcinoma
Source: Front Genet. 2020 Apr 28;11:413. doi: 10.3389/fgene.2020.00413 (PMC7198905; doi:10.3389/fgene.2020.00413)
Supplement: Supplementary file 3 [file Table_3.DOCX]

**Supplementary Table 3.** The primer sequences of 6 lncRNAs, 4 PCGs, and GAPDH.

| name | Primer Sequence |
| --- | --- |
| LINC02154 | forward 5′-GGTTGAGGAACTGTGCCTTGGAG-3′ |
|  | reverse 5′-GCCGGAGTTGCAGTGATGGAC-3′ |
| LINC00528 | forward 5′-AGCGTCCTTGAGGAGTCCAGTTC-3′ |
|  | reverse 5′-CAAGCACAGTCACGTTCTGAGGTC-3′ |
| SPRY4-AS1 | forward 5′-CACCAGCACCACCACCGAATG-3′ |
|  | reverse 5′-CCATTCCAGTGGCAGGCTCTTAAC-3′ |
| TTTY14 | forward 5′-GCCAATGGCTGTGACGGATAAGG-3′ |
|  | reverse 5′-ACCTGTTGCGGAGGACTACTGAG-3′ |
| LNCSRLR | forward 5′-CACTGTTCCAGGCACCAAGG-3′ |
|  | reverse 5′-TGTCGCCAAAGAAGAGAACAGG-3′ |
| KLHL7-DT | forward 5′-GAGCAGTAGGTCTCAACAGTGGAC-3′ |
|  | reverse 5′-CAACGATACCTAGCTGGCAGCAC-3′ |
| STC2 | forward 5′-TCATCAAAGACGCCTTGAAATG-3′ |
|  | reverse 5′-CAGCAAGTCCTTGAAATGGATC-3′ |
| TSPAN9 | forward 5′-TCGTCCTGTTGGTCATCCTCCTAG-3′ |
|  | reverse 5′-TTGGCGTTCTCGTTCACCTTGTC-3′ |
| SMS | forward 5′-TGGACCTTCAGAGTTATGATGG-3′ |
|  | reverse 5′-CCAGTACTGTCCTGACTCAATT-3′ |
| TCEA3 | forward 5′-CGGACGATGATTACAAGGACTA-3′ |
|  | reverse 5′-GCTCTTGAGCTCTTGGTAGATA-3′ |
| GAPDH | forward 5′-AAGGTGAAGGTCGGAGTCAA-3′ |
|  | reverse 5′-AATGAAGGGGTCATTGATGG-3′ |
