# Supplementary material for: The Prognostic Signature and Potential Target Genes of Six Long Non-coding RNA in Laryngeal Squamous Cell Carcinoma
Source: Front Genet. 2020 Apr 28;11:413. doi: 10.3389/fgene.2020.00413 (PMC7198905; doi:10.3389/fgene.2020.00413)
Supplement: Supplementary file 6 [file Table_6.DOCX]

**Supplementary Table 6.** Chi-square test to explore the correlation between 6 lncRNAs and clinicopathological characteristics of LSCC patients.

| Characteristics | LINC02154 | | | LINC00528 | | | SPRY4-AS1 | | | TTTY14 | | | LNCSRLR | | | KLHL7-DT | | |
| --- | --- | --- | --- | --- | --- | --- | --- | --- | --- | --- | --- | --- | --- | --- | --- | --- | --- | --- |
|  | Low | High | P value | Low | High | P value | Low | High | P value | Low | High | P value | Low | High | P value | Low | High | P value |
| Age at initial diagnosis | | | |  |  |  |  |  |  |  |  |  |  |  |  |  |  |  |
| <60 | 20 | 18 | 0.740 | 20 | 18 | 0.740 | 19 | 19 | 0.945 | 19 | 19 | 0.945 | 15 | 23 | 0.095 | 22 | 16 | 0.258 |
| >=60 | 36 | 37 |  | 36 | 37 |  | 37 | 36 |  | 37 | 36 |  | 41 | 32 |  | 34 | 39 |  |
| Gender |  |  |  |  |  |  |  |  |  |  |  |  |  |  |  |  |  |  |
| Male | 47 | 44 | 0.590 | 47 | 44 | 0.590 | 47 | 44 | 0.590 | 37 | 54 | <0.001*** | 45 | 46 | 0.653 | 48 | 43 | 0.302 |
| Female | 9 | 11 |  | 9 | 11 |  | 9 | 11 |  | 19 | 1 |  | 11 | 9 |  | 8 | 12 |  |
| Histologic grade | | | |  |  |  |  |  |  |  |  |  |  |  |  |  |  |  |
| G1+G2 | 38 | 40 | 0.553 | 40 | 38 | 0.782 | 35 | 43 | 0.114 | 40 | 38 | 0.553 | 37 | 41 | 0.304 | 40 | 38 | 0.720 |
| G3 | 16 | 13 |  | 14 | 15 |  | 18 | 11 |  | 13 | 16 |  | 17 | 12 |  | 16 | 13 |  |
| Gx | 2 | 2 |  | 2 | 2 |  | 3 | 1 |  | 3 | 1 |  | 2 | 2 |  | 0 | 4 |  |
| Clinical stage |  |  |  |  |  |  |  |  |  |  |  |  |  |  |  |  |  |  |
| I+II+III | 14 | 11 | 0.564 | 12 | 13 | 0.816 | 19 | 6 | 0.002** | 11 | 14 | 0.564 | 17 | 8 | 0.019* | 9 | 16 | 0.080 |
| IVA+IVB+IVC | 35 | 36 |  | 36 | 35 |  | 28 | 43 |  | 36 | 35 |  | 29 | 42 |  | 40 | 31 |  |
| NA | 7 | 8 |  | 8 | 7 |  | 9 | 6 |  | 9 | 6 |  | 10 | 5 |  | 7 | 8 |  |
| T stage |  |  |  |  |  |  |  |  |  |  |  |  |  |  |  |  |  |  |
| T1+T2 | 9 | 10 | 0.798 | 4 | 15 | 0.007** | 13 | 6 | 0.074 | 10 | 9 | 0.650 | 10 | 9 | 0.650 | 5 | 14 | 0.016* |
| T3+T4a | 40 | 39 |  | 44 | 35 |  | 36 | 43 |  | 37 | 42 |  | 37 | 42 |  | 45 | 34 |  |
| Tx | 5 | 6 |  | 7 | 4 |  | 6 | 5 |  | 8 | 3 |  | 7 | 4 |  | 5 | 6 |  |
| NA | 2 | 0 |  | 1 | 1 |  | 1 | 1 |  | 1 | 1 |  | 2 | 0 |  | 1 | 1 |  |
| N stage |  |  |  |  |  |  |  |  |  |  |  |  |  |  |  |  |  |  |
| N0 | 16 | 24 | 0.040* | 17 | 23 | 0.594 | 24 | 16 | 0.134 | 16 | 24 | 0.256 | 21 | 19 | 0.247 | 21 | 19 | 0.812 |
| N1-3 | 32 | 20 |  | 25 | 27 |  | 23 | 29 |  | 27 | 25 |  | 21 | 31 |  | 26 | 26 |  |
| Nx | 6 | 11 |  | 13 | 4 |  | 8 | 9 |  | 12 | 5 |  | 12 | 5 |  | 8 | 9 |  |
| NA | 2 | 0 |  | 1 | 1 |  | 1 | 1 |  | 1 | 1 |  | 2 | 0 |  | 1 | 1 |  |
| M stage |  |  |  |  |  |  |  |  |  |  |  |  |  |  |  |  |  |  |
| M0 | 20 | 20 | 1.000 | 19 | 21 | 1.000 | 20 | 20 | 1.000 | 20 | 20 | 1.000 | 21 | 19 | 1.000 | 26 | 14 | 0.366 |
| M1 | 1 | 0 |  | 0 | 1 |  | 1 | 0 |  | 0 | 1 |  | 1 | 0 |  | 0 | 1 |  |
| Mx | 3 | 5 |  | 7 | 1 |  | 3 | 5 |  | 5 | 3 |  | 3 | 5 |  | 5 | 3 |  |
| NA | 32 | 30 |  | 30 | 32 |  | 32 | 30 |  | 31 | 31 |  | 31 | 31 |  | 25 | 37 |  |
| Alcohol history | | | |  |  |  |  |  |  |  |  |  |  |  |  |  |  |  |
| Yes | 39 | 31 | 0.141 | 32 | 38 | 0.184 | 35 | 35 | 0.898 | 35 | 35 | 0.700 | 34 | 36 | 0.598 | 36 | 34 | 0.786 |
| No | 16 | 23 |  | 23 | 16 |  | 20 | 19 |  | 21 | 18 |  | 21 | 18 |  | 19 | 20 |  |
| NA | 1 | 1 |  | 1 | 1 |  | 1 | 1 |  | 0 | 2 |  | 1 | 1 |  | 1 | 1 |  |
| Smoking history | | | |  |  |  |  |  |  |  |  |  |  |  |  |  |  |  |
| Yes | 26 | 29 | 0.439 | 27 | 28 | 0.997 | 24 | 31 | 0.178 | 24 | 31 | 0.250 | 29 | 26 | 0.564 | 27 | 28 | 0.997 |
| No | 29 | 24 |  | 26 | 27 |  | 30 | 23 |  | 29 | 24 |  | 25 | 28 |  | 26 | 27 |  |
| NA | 1 | 2 |  | 3 | 0 |  | 2 | 1 |  | 3 | 0 |  | 2 | 1 |  | 3 | 0 |  |

NA, Not Applicable. *P < 0.05, **P < 0.01, ***P < 0.001.
